# Chemometric Discrimination of Korean and Chinese Kimchi Using Untargeted Metabolomics

**DOI:** 10.3390/metabo15100640

**Published:** 2025-09-25

**Authors:** Quynh-An Nguyen, Dong-Shin Kim, Hyo-Dong Kim, Kyu-Bin Kim, Kyung-Sik Ham, Yonghoon Lee, Hyun-Jin Kim

**Affiliations:** 1 Division of Applied Life Sciences (BK21 Four), Gyeongsang National University, 501 Jinjudaero, Jinju 52828, Gyeongsang, Republic of Korea; foodchem@gnu.ac.kr (Q.-A.N.); mnb9758@gnu.ac.kr (H.-D.K.); rbqls8155@gnu.ar.kr (K.-B.K.); 2 National Institute of Horticultural and Herbal Science, Rural Development Administration, Wanju 55365, Jeonbuk, Republic of Korea; dskim3309@korea.kr; 3 Department of Food Engineering, Mokpo National University, Muan 58554, Jeonnam, Republic of Korea; ksham@mnuac.kr; 4 Department of Chemistry, Mokpo National University, Muan 58554, Jeonnam, Republic of Korea; yhlee@mokpo.ac.kr; 5 Institute of Agriculture and Life Science, Department of Food Science & Technology, Gyeongsang National University, 501 Jinjudaero, Jinju 52828, Gyeongsang, Republic of Korea

**Keywords:** kimchi, metabolomics, chemometric origin discrimination, GC-MS, UPLC-Q-TOF MS

## Abstract

**Background/Objectives:** Kimchi has gained global recognition for its unique taste and health benefits, but its quality is totally different according to its geographical origin of materials and production methods. **Methods:** In this study, differences between Korean (53 samples) and Chinese kimchi (72 samples) were investigated through comprehensive metabolomic analysis using gas chromatography–mass spectrometry (GC-MS) and ultra-performance liquid chromatography–quadrupole time-of-flight mass spectrometry (UPLC-Q-TOF MS). **Results:** Multivariate statistical analyses revealed a clear separation between the two groups. Thirty-four metabolites contributing to the separation were identified. Korean kimchi was enriched in sucrose, quinic acid, sinapic acid derivatives, rutin, capsicosin, and capsianoside, while Chinese kimchi contained higher levels of trihydroxy octadecenoic acid, 2-hydroxypalmitic acid, pinellic acid, maltose, glucuronic acid, and corchorifatty acid F. In particular, the univariate Bayesianlogistic regression analysis revealed that among these metabolites, rutin, capsicosin derivatives, and sinapic acid derivatives showed strong potential as origin-discriminant markers of kimchi, providing insights into how these metabolites influence its nutritional and sensory properties. **Conclusions:** These compositional differences may be attributed to variations in raw materials and production methods of kimchi.

## 1. Introduction

The origin and authenticity of food products are a critical concern in the modern food industry. Mislabeling and adulteration can pose significant risks to public health, economic stability, and the expected nutritional and health benefits by introducing potential allergens, toxins, and low-quality ingredients [1,2]. This problem is exacerbated in the global market, where complex supply chains can obscure the origin and ingredients of food materials [1,2]. Therefore, the development of advanced analytical methods is essential to ensure the quality of food materials and to verify their origin. Conventional methods for food authentication have included a range of techniques, from sensory evaluations to advanced molecular analyses [3]. Sensory evaluation using electronic tongue and nose as well as non-destructive testing methods like infrared spectrometry can offer a faster approach, but their specificity is often limited [4]. DNA-based techniques, such as polymerase chain reaction (PCR), target specific genetic markers to confirm the identity of food ingredients [5], while targeted analytical methods of chemical profiles focus on specific compounds [6]. Physical elemental analyses using inductively coupled plasma mass spectrometry (ICP-MS) and isotope ratio mass spectrometry (IRMS) can also provide detailed information for food authentication but are often limited to specific types of adulteration [6].

In addition to these analytical methods, metabolomics, the simultaneous and comprehensive analysis of numerous metabolites in biological systems, is widely used as an informative, discriminative, and predictive technique for assessing food quality, safety, and geographical origin [7,8]. In particular, it has proven to be powerful and effective for determining the geographical differentiation of food products by profiling metabolic signatures. This approach has been applied to various foods, including honey [9], wine [10], olive oils [11], red wines [12], coffee [13], and seafood [14], vegetables, and fruits. Additionally, the study of metabolite profiling in fermented foods is used to observe metabolite changes during fermentation and predict the sensory and nutritional quality of the final food materials, such as meju [15], doenjang [16], fermented soymilk [17], and yogurt [18].

Kimchi, known as one of the healthiest foods, is gaining global popularity due to its distinctive taste and potential health benefits [19]. However, with the growing size of the kimchi market size, a proliferation of kimchi products from various origins has raised concerns about their geographical origin and production methods. Although several metabolomic studies have investigated kimchi [20,21], few have focused on differentiating products based on their production regions. Several previous studies have investigated the geographical discrimination of kimchi, including NMR-based metabolomics [22], elemental profiling [23], and ICP-OES/ICP-MS with multivariate statistics [24]. Nevertheless, applications of combined GC-MS and UPLC-QTOF MS with machine-learning approaches remain limited.

Therefore, in this study, gas chromatography (GC)-MS and ultra-performance liquid chromatography-quadrupole-time-of-flight (UPLC-Q-TOF) MS were employed to analyze untargeted metabolites from 125 commercial kimchi samples produced in two different countries. Multivariate statistical analysis was conducted to identify discriminative metabolites associated with regional differences, in order to establish candidate markers for the chemometric classification of kimchi according to production region.

## 2. Materials and Methods

### 2.1. Kimchi Sample

A total of 125 cabbage kimchi samples were collected by the National Agricultural Products Quality Management Service of South Korea in 2021. Among them, 72 samples were imported from China, and 53 samples produced in Korea and obtained from local markets. All kimchi samples were collected within 10 days of production. All samples were washed with distilled water, freeze-dried, and ground into powder.

### 2.2. pH, Salinity, and Titratable Acidity

The physicochemical properties of the kimchi samples, including pH, salinity, and titratable acidity (TA), were subsequently analyzed. For these measurements, 0.1 g of sample powder was mixed with 0.9 mL of distilled water. Salinity was measured with a salt meter (PAL-SALT, Atago, Minato, Japan), while pH and TA were determined using a pH meter (HI 2215, HANNA Instruments, Woonsocket, RI, USA).

### 2.3. GC-MS Analysis

The lyophilized kimchi samples were homogenized in 80% aqueous methanol containing dicyclohexyl phthalate as an internal standard (IS). After centrifugation, the supernatant was completely dried. The dried residues were re-dissolved in 70 μL of methoxyamine hydrochloride in pyridine (20 mg/mL) and incubated at 37 °C for 90 min. The samples were derivatized by adding 70 μL of N,O-bis(trimethylsilyl)trifluoroacetamide with 1% trimethylchlorosilane and incubated at 70 °C for 30 min. The derivatized sample was injected into a GC-2010 plus system (Shimadzu Corp., Kyoto, Japan) equipped with a DB-5ms capillary column (30 m × 0.25 mm, 0.25 μm, Agilent J&W column; Agilent Technologies, Santa Clara, CA, USA) using a split ratio of 1:50. The injector temperature was set to 200 °C, and the carrier gas flow rate was 1 mL/min. The oven was held at 70 °C for 2 min, ramped to 210 °C at 7 °C/min, then to 320 °C at 10 °C/min, and finally held at 320 °C for 7 min. The eluents were detected using a GCMS-TQ 8030 MS system (Shimadzu Corp.) with electron ionization at 70 eV. Ion source and interface temperatures were set to 230 °C and 280 °C, respectively. Data were acquired in full-scan mode over the m/z range of 45–550. A quality control (QC) sample, prepared by mixing aliquots of all samples, was injected once after every sample set [25].

### 2.4. UPLC-Q-TOF MS Analysis

Metabolites were extracted from lyophilized kimchi samples by homogenization with 80% methanol containing zidovudine as an IS. After centrifugation, the supernatants were analyzed by UPLC-Q-TOF MS (Xevo™ G2-S, Waters, Milford, MA, USA) equipped with an Acquity UPLC BEH C18 column (2.1 mm × 100 mm, 1.7 μm; Waters). The column was equilibrated with 0.1% formic acid, and metabolites were eluted using a linear gradient up to 100% acetonitrile containing 0.1% formic acid. The eluted metabolites were analyzed using the Q-TOF MS system in negative electrospray ionization (ESI) mode with a capillary voltage of 3 kV and a sampling cone voltage of 40 V. Leucine-enkephalin was used as a lock mass reference. A QC sample prepared by pooling aliquots of all samples was analyzed between every sample set. MS/MS spectra were acquired using a collision energy ramp from 10 to 30 eV [25].

### 2.5. Data Processing

The peak intensities obtained by GC-MS were aligned based on the retention time and normalized to the IS. The MS dataset analyzed by UPLC-Q-TOF MS was collected, aligned using MarkerLynx software ver. 4.2. (Waters), and normalized to the IS. The metabolites analyzed by UPLC-Q-TOF MS were identified using Chemspider databases implemented in UNIFI (Waters), and in-house database constructed with commercially available authentic standards, and MS/MS spectra. The metabolites analyzed by GC/MS were identified by comparing retention indices (RIs) calculated using C8-C40 *n*-alkanes with GC-MS databases (NIST 11 and Wiley 9 mass spectral libraries).

### 2.6. Predictive Modeling of Kimchi Biomarkers

To evaluate the predictive performance of the identified kimchi biomarkers, the dataset was randomly partitioned into a training set (70%) and a test set (30%). Logistic regression models were trained with a maximum of 500 iterations and a fixed random state (123) to ensure convergence and reproducibility. Model performance was further assessed using confusion matrix–based metrics (accuracy, sensitivity, specificity) on the held-out test set to complement ROC analysis. Univariate Bayesian logistic regression models were fitted on the training data to assess the discriminatory ability of each biomarker individually. The predictive performance of each model was then evaluated on the held-out test set. Receiver operating characteristic (ROC) curve analysis was performed to visualize the discriminatory power of each biomarker, and the area under the curve (AUC) was calculated. Biomarkers with higher AUC values were considered to have greater discriminatory potential.

### 2.7. Statistical Analysis

Multivariate statistical analysis of the MS data was performed using SIMCA-P+ version 16.0.1 (Umetrics, Umeå, Sweden). Partial least squares discriminant analysis (PLS-DA) was used to visualize the differences among sample groups, and was selected because it provides a robust and reliable discrimination model for metabolomics data. Metabolite intensities were statistically analyzed to identify metabolites contributing to the differences among sample groups by two-way analysis of variance (ANOVA) followed by a *t*-test (*p* < 0.05) using SPSS 27.0 (SPSS Inc., Chicago, IL, USA). Moreover, pH, salinity, and acidity were also statistically analyzed using a *t*-test (*p* < 0.05). Normality was not explicitly tested; however, given the relatively large sample sizes (Korean kimchi: *n* = 53; Chinese kimchi: *n* = 72), parametric methods are considered robust to moderate deviations from normality [26].

## 3. Results and Discussion

### 3.1. Physicochemical Properties of Kimchi Samples

The pH, salinity, and TA of 125 kimchi samples produced in two countries with geographical differences were approximately 3.2–3.5, 4.9, and 0.22%, respectively (Table 1). However, no statistically significant differences were observed between the kimchi samples. In kimchi fermentation, pH typically decreases rapidly from around 5–6 to 3–4 within the first few days and then remains relatively stable. In contrast, TA, which is below 0.2% at the initial stage, continues to increase throughout fermentation, depending on conditions such as temperature and microbial activity [21,27]. In this study, the observed pH combined with relatively low acidity suggests that the samples were at a comparable early-to-mid stage of fermentation across both countries [27].

### 3.2. Metabolomic Analysis

**Figure 1:**
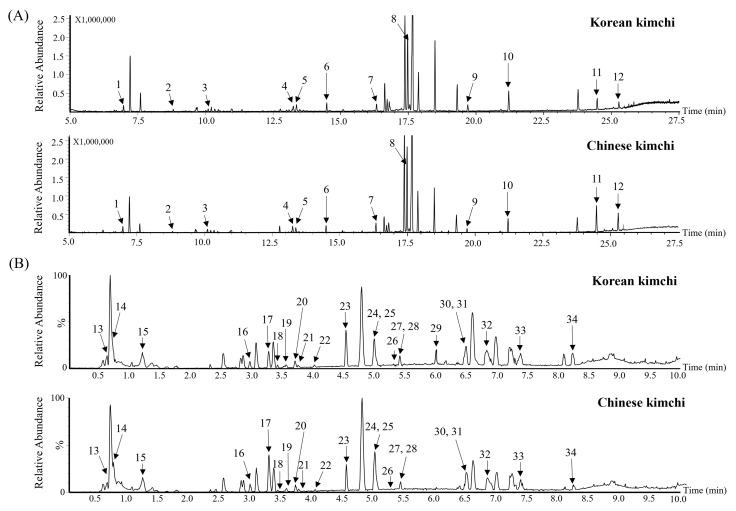
Representative chromatograms of global metabolites in washed kimchi obtained using GC-MS (**A**) and UPLC-Q-TOF MS (**B**). 1, alanine; 2, valine; 3, proline; 4, oxoproline; 5, 4-aminobutanoic acid; 6, glutamic acid; 7, glutamine; 8, glucose; 9, inositol; 10, stearic acid; 11, sucrose; 12, maltose; 13, glucuronic acid; 14, quinic acid; 15, pyroglutamic acid; 16, phenylsulphonyl isocyanate; 17, neoglucobrassicin; 18, rutin; 19, carlinoside; 20, sinapic acid; 21, disinapoyl sucrose; 22, tri sinapoyl gentibioside; 23, capsianoside; 24, pinellic acid; 25, corchorifattty acid F; 26, virescenoside R; 27, trihydroxy octadecenoic acid; 28, albocyline; 29, capsicosin; 30, lysophosphatidylethanolamine (18:3); 31, lysophosphatidylcholine (18:3); 32, peroxy linoleic acid; 33, lipopolysaccharide (21:0); 34, 2-hydroxypalmitic acid.

**Figure 2:**
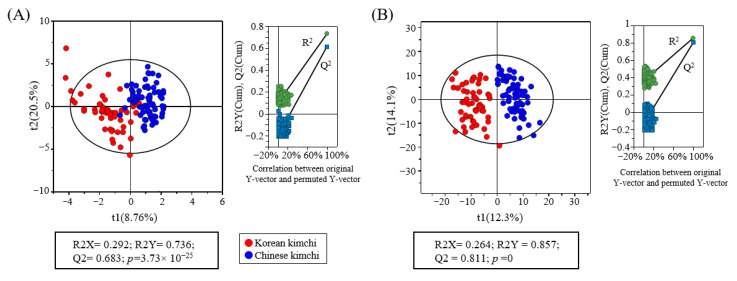
Partial least-squares discriminant analysis (PLS-DA) score plot of obtained kimchi metabolites analyzed using GC–MS (**A**) and UPLC-Q-TOF MS (**B**). The quality of PLS-DA models was evaluated by R2X, R2Y, Q2, and *p*-values, and validated by permutation test. R2X and R2Y indicate the goodness of fit, while Q2 reflects the predictive ability of the models.

The metabolite profiles of 125 samples (53 Korean and 72 Chinese kimchi samples) were analyzed using GC-MS and UPLC-Q-TOF MS (Figure 1). The acquired MS data were subjected to multivariate statistical analysis to visualize the differences between the two kimchi groups (Figure 2). The statistical parameters, including the goodness of fit (R2Y = 0.815 and 0.857), predictability (Q2 = 0.683 and 0.911), *p*-values (1.60 × 10^−30^ and 0), and cross-validation results from permutation tests (y-intercepts: GC-MS, R^2^ = 0.102 and Q^2^ = −0.200; UPLC-Q-TOF MS, R^2^ = 0.352 and Q^2^ = −0.136) determined via the permutation test, indicated that the PLS-DA models used in this study were statistically acceptable. The PLS-DA score plots showed that the two kimchi groups were significantly separated along t(1). To identify metabolites contributing to the PLS-DA plots separation and potential markers, the variable importance in projection (VIP) and *p*-values were calculated, and a total of 34 metabolites having VIP > 0.88 and *p* < 0.05 were identified (Table 2 and Table 3). Similar metabolites, such as sinapic acid derivatives, glucosinolates, rutin, and GABA, have also been reported in previous kimchi and kimchi cabbage metabolomics studies [25,28].

### 3.3. Relative Abundance of Identified Metabolites

**Figure 3:**
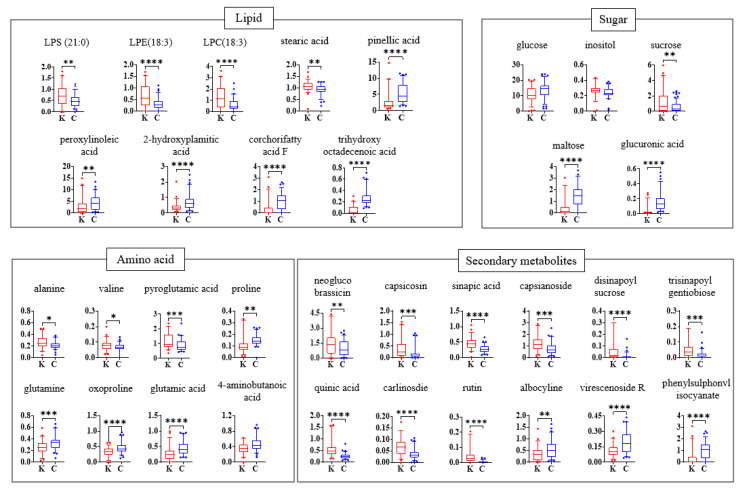
Comparison of relative abundance of kimchi metabolites. Box plots present the relative abundance of metabolites analyzed by GC–MS and UPLC-Q-TOF MS. The y-axis shows the normalized chromatogram intensity and x-axis shows the kimchi samples. K, Korean kimchi; C, Chinese kimchi. LPS, lipopolysaccharide; LPE, lysophosphatidylethanolamine; LPC, lysophosphatidylcholine. Statistical significance was determined by *t*-tests at *p* < 0.05 (*), *p* < 0.01 (**), *p* < 0.001 (***), and *p* < 0.0001 (****).

Based on identified metabolites, significant differences in metabolite composition between the two kimchi groups were observed (Figure 3). The comparison of relative abundance revealed that the two kimchi groups exhibited markedly different metabolite distribution patterns in lipids, sugars, amino acids, and secondary metabolites.

Among these metabolites, several sugars showed distinct differences in abundance between Korean and Chinese kimchi. Sucrose was 2.20-fold more abundant in Korean kimchi compared to Chinese kimchi, whereas glucose, maltose, and glucuronic acid were 1.29-, 3.20-, and 4.56-fold higher in Chinese kimchi.

Sucrose is typically hydrolyzed in the early stage of kimchi fermentation, followed by the gradual reduction in fructose and glucose, while disaccharides such as maltose increase [21]. However, in this study, Korean kimchi showed higher levels of sucrose than Chinese kimchi. This may reflect the use of sugar-containing ingredients in some commercial products, rather than differences in fermentation extent [29,30].

In contrast, the higher levels of glucose, maltose, and glucuronic acid in Chinese kimchi suggest active degradation of starch and plant cell wall polysaccharides. These metabolites may originate from starchy ingredients such as glutinous rice or wheat flour paste, which are often added during kimchi preparation. In particular, the significant increase in maltose, a typical product of starch hydrolysis, implies the involvement of amylolytic enzymes. Glucuronic acid, a constituent of hemicellulose and pectin, may be released during their microbial degradation [31] suggesting possible differences in ingredient composition or enzymatic activity between the two groups.

Regarding amino acids, the levels of alanine, valine, oxoproline, and pyroglutamic acid were approximately 1.2–1.4 times lower in Chinese kimchi than in Korean kimchi, whereas proline, glutamine, 4-aminobutanoic acid (GABA), and glutamic acid were approximately 1.2–1.4 times higher. This difference can be explained by the metabolic activities of bacteria involved in kimchi fermentation. Lactic acid bacteria (LAB) such as *Lactobacillus plantarum* and *Leuconostoc mesenteroides* are responsible for utilizing alanine and valine early in the fermentation process, resulting in their lower concentrations. These bacteria convert these amino acids into flavor-enhancing metabolites, which are used up quickly during fermentation, particularly in Chinese kimchi, where fermentation may proceed more rapidly [32,33]. In contrast, proline, glutamine, and glutamic acid tend to accumulate more in Chinese kimchi, potentially due to microbial activities or shorter fermentation times that limit the breakdown of these amino acids [34]. These amino acids, especially glutamic acid and glutamine, contribute to the umami flavor, and their higher levels indicate that the fermentation process in Chinese kimchi favors the retention of these flavor-enhancing compounds [35]. Importantly, GABA, a bioactive compound with health-promoting effects, is produced by LAB (e.g., *Lactobacillus plantarum*) through the decarboxylation of glutamic acid [36]. Its elevated levels in Chinese kimchi suggest that GABA-producing LAB strains are active or that fermentation conditions in Chinese kimchi favor increased GABA accumulation [37].

Differences in lipid profiles also contribute substantially to the discrimination between Korean and Chinese kimchi. LPE (18:3) and LPC (18:3), phospholipids associated with cell membrane processes, were detected at approximately two-fold lower concentrations in Chinese kimchi (Figure 3). Conversely, several fatty acids, including trihydroxy octadecadienoic acid, pinellic acid, peroxylinoleic acid, 2-hydroxypalmitic acid, and corchorifatty acid F, were detected at higher concentrations in Chinese kimchi, with fold increases of 4.70, 2.00, 1.54, 1.83, and 3.20, respectively (Table 3). The observed differences in lipid profiles between Korean and Chinese kimchi may be attributable to multiple factors, including the lipid composition of raw materials, fermentation conditions, and microbial communities [38]. Dominant LAB and their associated lipolytic and phospholipase activities can alter phospholipid degradation and fatty acid oxidation, thereby shaping the distinct lipid profiles of each regional product [29].

Following the differences observed in primary metabolites such as sugars, amino acids, and lipids, the secondary metabolomic profiling also revealed significant distinctions between Korean and Chinese kimchi. In particular, the levels of quinic acid, neoglucobrassicin, capsicosin, sinapic acid, capsianoside, disinapoyl sucrose, trisinapoyl gentiobiose, carlinoside, and rutin were 2.18-, 1.56-, 2.23-, 1.64-, 1.55-, 6.27-, 2.58-, 1.83-, and 7.45-fold higher in Korean kimchi, respectively, whereas the levels of virescenoside R and phenylsulphonyl isocyanate were 1.63- and 2.68-fold higher in Chinese kimchi. Capsicosin and capsianoside are capsaicinoid-related metabolites derived from Capsicum annuum, contributing to pungency as well as antimicrobial and antioxidant activities [39]. Their elevated abundance in Korean kimchi may reflect the use of chili cultivars with a higher genetic capacity for capsaicinoid biosynthesis, differences in production methods, and possible environmental or geographical influences [40]. Previous studies have also reported that elevated levels of these metabolites can influence kimchi fermentation by altering the microbial community [41]. Higher capsaicinoid concentrations have been associated with a relative increase in capsaicinoid-tolerant lactic acid bacteria such as *Lactobacillus sakei*, while reducing the abundance of *Leuconostoc gelidum*. Such shifts may modify fermentation kinetics and ultimately affect the overall quality of kimchi [41,42]. Similarly, higher levels of quinic acid, neoglucobrassicin, sinapic acid derivatives, carlinoside, rutin, and isoeruboside B in Korean kimchi are also likely shaped by cultivar characteristics and production methods, with possible contributions from environmental and geographical factors [43]. Notably, sinapic acid derivatives are among the major phenolic constituents of kimchi cabbage, the primary vegetable in kimchi, and their higher levels in Korean kimchi may reflect compositional differences in the kimchi cabbage used [28,44]. These metabolites are reported to possess antioxidant, anti-inflammatory, and metabolic health–modulating activities and may contribute to both the functional properties and sensory characteristics of kimchi [45,46]. The differences are consistent with origin-associated production methods, although ingredients, microbiota, and production conditions were not directly assessed in this study.

### 3.4. Discriminative Performance of Key Metabolites for Kimchi Origin Discrimination

**Figure 4:**
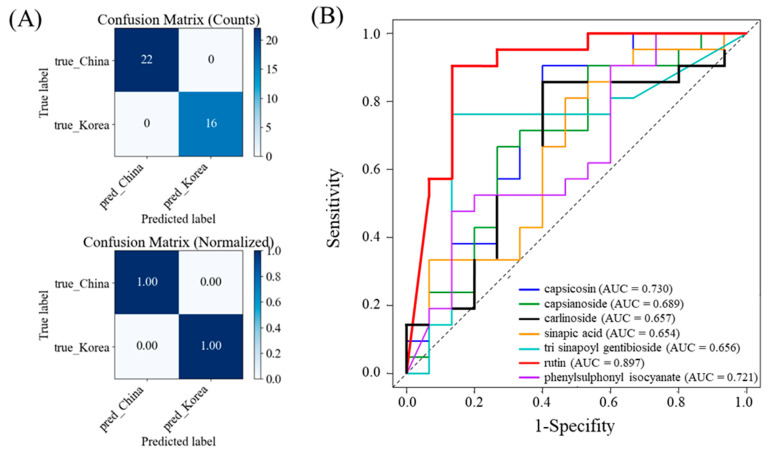
The confusion matrices (**A**) and receiver operating characteristic (ROC) curves (**B**) illustrating the predictive performance of the combined model based on major kimchi metabolites discriminating between Korean and Chinese samples. AUC, area under the curve.

To evaluate the discriminative ability of major metabolites, logistic regression and ROC curve analyses were performed in R (Figure 4). The logistic regression model demonstrated a complete classification accuracy of 100% within the test dataset, accurately identifying all samples from the Chinese (*n* = 22) and the Korean (*n* = 16) (Figure 4A). To complement the AUC values, classification performance was evaluated at the optimal ROC threshold (Table S1); across the top markers, accuracy ranged from 0.639 to 0.889, sensitivity from 0.476 to 0.905, and specificity from 0.533 to 0.867. Rutin showed the highest classification performance with AUC of 0.897, indicating excellent ability to distinguish Korean and Chinese kimchi samples. Capsicosin (AUC = 0.730) and phenylsulphonyl isocyanate (AUC = 0.721) also demonstrated strong classification performance. Carlinoside (AUC = 0.670), capsianoside (AUC = 0.670), trisinapoyl gentiobiose (AUC = 0.656), and sinapic acid (AUC = 0.654) showed moderate discrimination capacity. The superior performance of rutin, capsicosin, and phenylsulphonyl isocyanate highlights their potential as key metabolites for distinguishing kimchi by origin. In particular, rutin showed excellent classification accuracy, suggesting that it could serve as a reliable single marker. Nevertheless, metabolites such as capsicosin and sinapic acid derivatives, despite lower individual AUCs, may contribute synergistically to multi-marker classification models, thereby enhancing robustness and predictive reliability [47,48].

## 4. Conclusions

This study demonstrates that the comparative metabolomic profiling of Korean and Chinese kimchi revealed distinct signatures in primary and secondary metabolites through GC-MS and UPLC-Q-TOF MS analyses. Korean kimchi was enriched in sucrose, phenolic compounds, and capsaicinoid-related compounds, including quinic acid, sinapic acid derivatives, rutin, capsicosin, and capsianoside. In contrast, Chinese kimchi contained higher levels of trihydroxy octadecenoic acid, 2-hydroxypalmitic acid, pinellic acid, maltose, glucuronic acid, and A corchorifatty acid F. Among these metabolites, metabolites such as rutin, capsicosin derivatives, and sinapic acid derivatives showed strong potential as origin-discriminant markers, reflecting the influence of raw materials, fermentation practices, and environmental factors on kimchi composition. These markers provide reliable tools for authenticating kimchi origin and enhance understanding of how geographic variation, as well as differences in recipe, ingredients, and production processes, may affect its nutritional and sensory qualities.

## Figures and Tables

**Table 1:** Physicochemical characteristics of Korean kimchi and Chinese kimchi samples.

	Korean Kimchi	Chinese Kimchi
pH	4.905 ± 0.533	4.886 ± 0.524
salinity (%)	3.454 ± 0.763	3.153 ± 0.665 *
TA (% lactic acid)	0.219 ± 0.059	0.218 ± 0.055

Values are expressed as mean ± SD (Korean kimchi, *n* = 53; Chinese kimchi, *n* = 72). * *p* < 0.05 by Student’s *t*-test.

**Table 2:** Identification of major metabolites contributing the separation between sample groups by GC-MS.

RT (min)	Compound	RI	VIP	*p*-Value	Fold Change (vs. K)
6.98	alanine	1097	1.06	5.43 × 10^−3^	−1.23
8.82	valine	1210	0.97	1.14 × 10^−2^	−1.21
10.10	proline	1292	1.17	7.24 × 10^−3^	1.25
13.25	oxoproline	1516	0.88	1.20 × 10^−2^	−1.19
13.37	4-aminobutanoic acid	1525	1.65	6.34 × 10^−5^	1.33
14.49	glutamic acid	1612	1.86	5.82 × 10^−6^	1.74
16.34	glutamine	1766	1.10	9.18 × 10^−3^	1.26
17.67	glucose	1843	1.45	3.73 × 10^−2^	1.29
19.70	inositol	1981	0.88	6.45 × 10^−2^	−1.14
21.22	stearic acid	2132	1.14	2.30 × 10^−4^	−1.12
24.48	sucrose	2609	1.24	4.27 × 10^−3^	−2.20
25.28	maltose	2723	2.23	4.35 × 10^−9^	3.20

RT, retention time; RI, retention index; VIP, variable importance in projection; K, Korean kimchi. *p*-values were calculated by Duncan’s test.

**Table 3:** Identification of major metabolites contributing the separation between sample groups by UPLC-Q-TOF MS.

RT (min)	Compound	Exact Mass (M-H)	MS Fragments	VIP	*p*-Value	Fold Change (vs. K)
0.69	glucuronic acid	193.035	113	1.68	1.19 × 10^−10^	4.56
0.74	quinic acid	191.055	179	1.68	1.25 × 10^−10^	−2.18
1.20	pyroglutamic acid	128.033	116	1.00	5.04 × 10^−5^	−1.38
2.93	phenylsulphonyl isocyanate	181.990	119	1.32	1.78 × 10^−6^	2.68
3.27	neoglucobrassicin	477.063	422, 609	0.85	3.00 × 10^−3^	−1.56
3.40	rutin	609.147	284	1.31	1.29 × 10^−6^	−7.45
3.52	carlinoside	579.137	399	1.39	3.36 × 10^−7^	−1.83
3.71	sinapic acid	223.060	149, 164,193	1.59	1.73 × 10^−9^	−1.64
3.85	disinapoyl sucrose	753.225	529, 205	1.08	9.54 × 10^−5^	−6.27
4.09	trisinapoyl gentiobiose	959.287	735, 529, 511, 205	1.03	1.92 × 10^−4^	−2.58
4.53	capsianoside	1129.536	937, 497, 343	1.06	2.70 × 10^−5^	−1.55
5.00	pinellic acid	329.231	211, 229, 171, 139	1.33	1.42 × 10^−6^	2.00
5.00	corchorifatty acid F	327.218	211, 171, 229, 291	1.65	4.12 × 10^−10^	3.20
5.24	virescenoside R	643.336	293, 481	1.17	2.07 × 10^−6^	1.63
5.33	trihydroxy octadecenoic acid	329.232	171, 201	2.16	8.03 × 10^−19^	4.70
5.41	albocyline	307.191	116, 329, 805	0.90	1.00 × 10^−3^	1.55
5.95	capsicosin	1287.595	915	1.05	3.32 × 10^−4^	−2.23
6.43	LPE (18:3)	474.262	277, 196	1.33	1.06 × 10^−6^	−2.04
6.45	LPC (18:3)	562.316	502, 277	1.23	8.31 × 10^−6^	−2.03
6.88	peroxy linoleic acid	295.226	277, 233	0.99	1.72 × 10^−10^	1.54
7.38	LPS (21:0)	566.348	281, 152, 506	0.88	2.00 × 10^−3^	−1.45
8.22	2-hydroxypalmitic acid	271.227	225, 255	1.13	5.81 × 10^−6^	1.83

RT, retention time; VIP, variable importance in projection; K, Korean kimchi; LPE, lysophosphatidylethanolamine; LPC, lysophosphatidylcholine; LPS, lipopolysaccharide. *p*-values were calculated by Duncan’s test.

## Data Availability

The original contributions presented in this study are included in the article/Supplementary Materials. Further inquiries can be directed to the corresponding author.

## Supplementary Materials

The following supporting information can be downloaded at https://www.mdpi.com/article/10.3390/metabo15100640/s1: Figure S1: Variable importance in projection (VIP) values of metabolites from the GC-MS-based PLS–DA model; Figure S2: Variable importance in projection (VIP) values of metabolites from the UPLC-Q-TOF MS based PLS–DA model; Table S1: Classification performance (accuracy, sensitivity, specificity) of the logistic regression model at optimal ROC thresholds for discriminating Korean vs. Chinese kimchi.
