# Supplementary material for: Chemometric Discrimination of Korean and Chinese Kimchi Using Untargeted Metabolomics
Source: Metabolites. 2025 Sep 25;15(10):640. doi: 10.3390/metabo15100640 (PMC12566599; doi:10.3390/metabo15100640)
Supplement: Supplementary file 1 [file metabolites-15-00640-s001.zip › metabolites-3861216-supplementary.pdf]

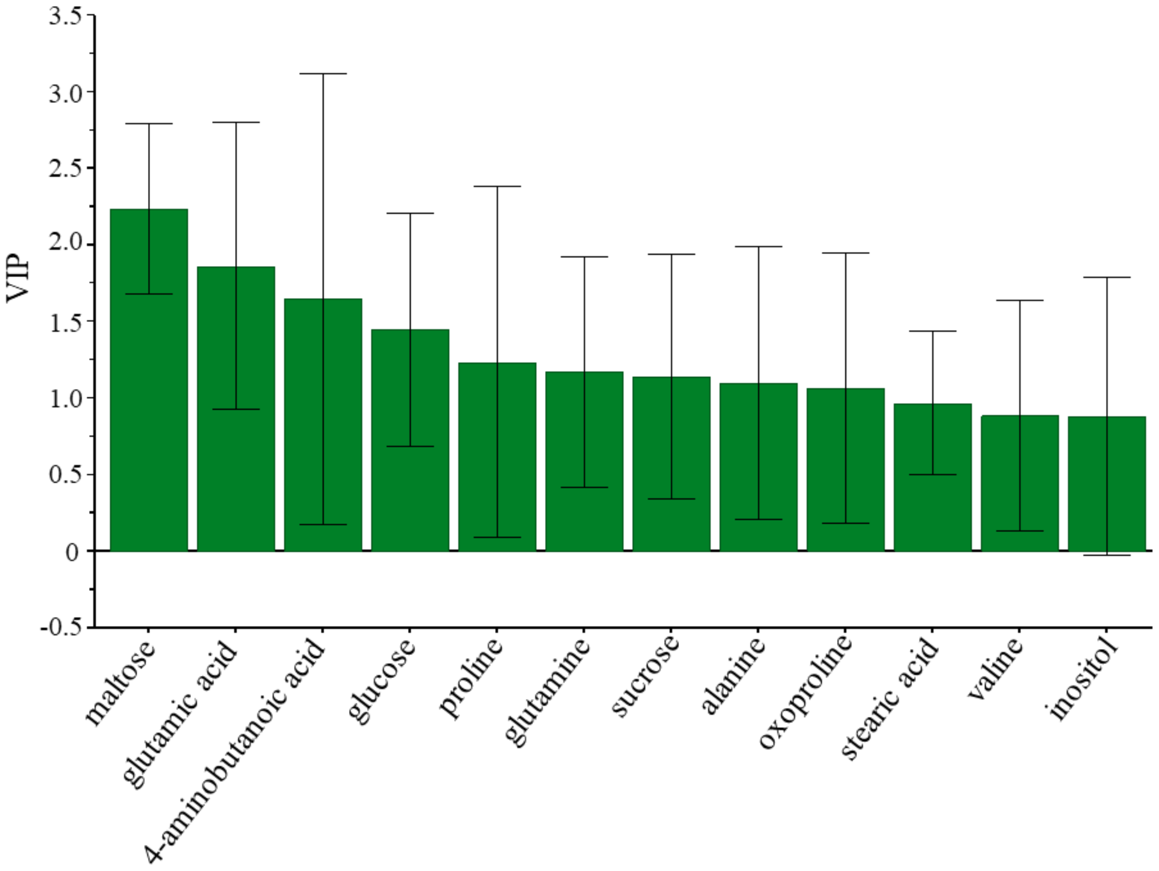

Fig. S1. Variable importance in projection (VIP) values of metabolites from the GC-MS-based PLS–DA model.

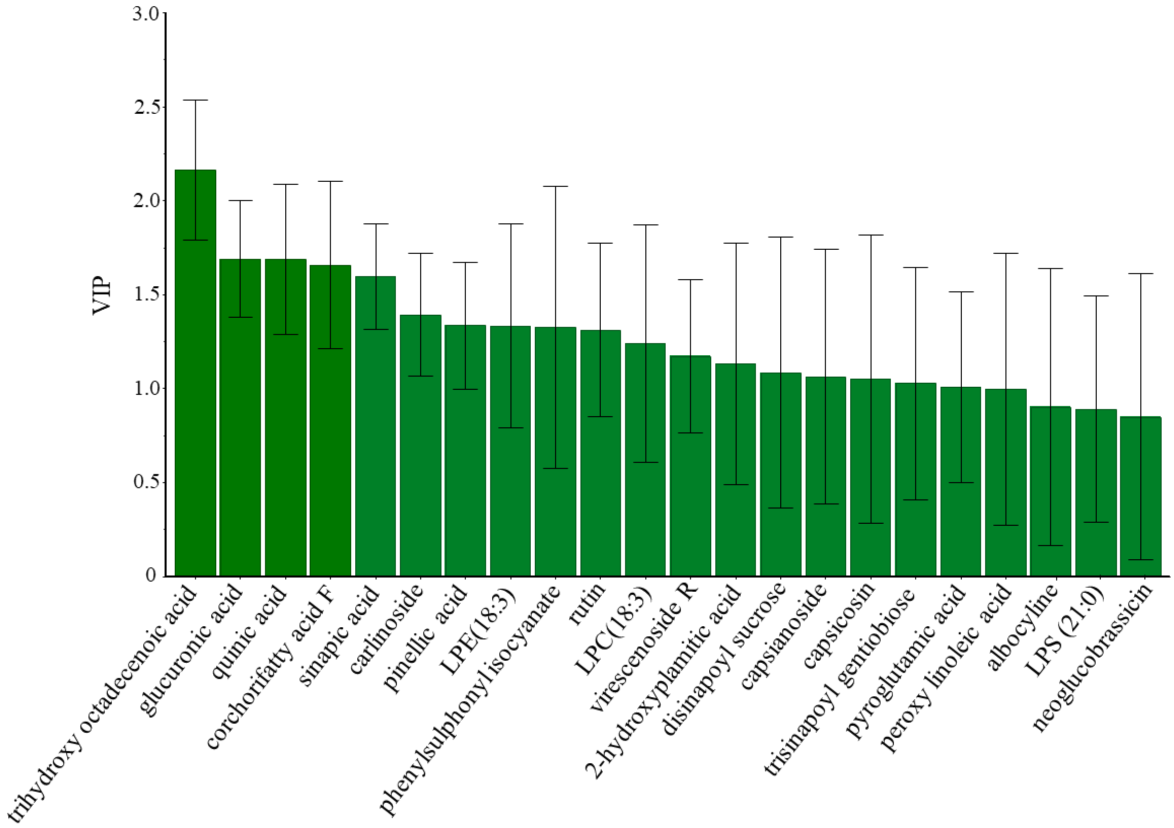

Fig. S2. Variable importance in projection (VIP) values of metabolites from the UPLC-Q-TOF MS -based PLS–DA model.

Table S1.

Classification performance (accuracy, sensitivity, specificity) of the logistic regression model at optimal ROC thresholds for discriminating Korean vs. Chinese kimchi.

Compound	Accuracy	Sensitivity	Specificity
capsicosin	0.778	0.905	0.600
phenylsulphonyl isocyanate	0.806	0.867	0.611
rutin	0.889	0.905	0.867
trisinapoyl gentiobiose	0.639	0.476	0.867
sinapic acid	0.694	0.810	0.533
capsianoside	0.694	0.714	0.667
carlinosdie	0.750	0.857	0.600
